# Rescue of Infectious Birnavirus from Recombinant Ribonucleoprotein Complexes

**DOI:** 10.1371/journal.pone.0087790

**Published:** 2014-01-30

**Authors:** Romy M. Dalton, José F. Rodríguez

**Affiliations:** Department of Molecular and Cellular Biology, Centro Nacional de Biotecnología-CSIC, Cantoblanco, Madrid, Spain; INRA, France

## Abstract

Birnaviruses are unconventional members of the icosahedral double-stranded (dsRNA) RNA virus group. The main differential birnavirus trait is the lack of the inner icosahedral transcriptional core, a ubiquitous structure conserved in all other icosahedral dsRNA viruses, that shelters the genome from cellular dsRNA sensors and provide the enzymatic machinery to produce and extrude mature messenger RNAs. In contrast, birnaviral particles enclose ribonucleoprotein (RNP) complexes formed by the genome segments, the dsRNA-binding VP3 polypeptide and the virus-encoded RNA polymerase (RdRp). The presence of RNPs suggests that the birnavirus replication program might exhibit significant differences with respect to those of prototypal dsRNA viruses. However, experimental evidences supporting this hypothesis are as yet scarce. Of particular relevance for the understanding of birnavirus replication is to determine whether RNPs act as intracellular capsid-independent transcriptional units. Our study was focused to answer this question using the infectious bursal disease virus (IBDV), the best characterized birnavirus, as model virus. Here, we describe the intracellular assembly of functional IBDV RNPs in the absence of the virus-encoded VP2 capsid polypeptide. Recombinant RNPs are generated upon coexpression of the IBDV VP1 and RdRp polypeptides and transfection of purified virus dsRNA. Presented data show that recombinant RNPs direct the expression of the IBDV polypeptide repertoire and the production of infectious virus in culture cells. Results described in this report constitute the first direct experimental evidence showing that birnaviral RNPs are intracellularly active in the absence of the virus capsid. This finding is consistent with presented data indicating that RNP formation precedes virus assembly in IBDV-infected cells, and supports the recently proposed IBDV replication model entailing the release of RNPs during the initial stages of the infection. Indeed, results presented here also support the previously proposed evolutionary connection between birnaviruses and positive-strand single-stranded RNA viruses.

## Introduction

Virus capsids are specifically designed to accurately deliver the viral genome to a correct destination within the host cell. In general terms, the genome of RNA viruses may be stored in the virus particle as: i) positive-strand (+) single-stranded (ss) RNA; ii) negative-strand (−) ssRNA; or iii) double-stranded (ds) RNA. Despite these genome differences, replication of RNA viruses invariably involves the formation of dsRNA molecules. This represents a major threat due to the presence of highly sophisticated host sensor proteins capable of triggering efficient antiviral responses [1]. +ssRNA virions lack virus-encoded RNA-dependent RNA polymerases (RdRp), and their gene expression is initiated by the release of functional virus-encoded mRNAs upon virus entry. These viruses assemble their RNA replication/transcription complexes in close association with membranous cell compartments that provide an effective shield against dsRNA sensors [2]. -ssRNA viruses carry their own RdRp and prevent the exposure of duplex RNA by protecting their genomic RNA with nucleocapsid proteins, and by assembling transcriptionally active ribonucleoprotein (RNP) complexes [3]. Finally, prototypical dsRNA viruses enclose their genomes in a highly conserved structure, the so-called T = 2 capsid or transcriptional core, that contains all the enzymatic machinery required to synthesize and extrude translatable mRNAs into the cytoplasm of the infected cell. T = 2 capsids from dsRNA viruses with life cycles including extracellular phases, usually enclosed by one or two additional capsid shells, are released into the cell endosome upon virus entry, and remain intact throughout the entire replication cycle. In these viruses, RNA replication is associated to virus morphogenesis and takes place inside assembling previrion particles, thus precluding the detection of the newly synthesized dsRNA and the subsequent activation of innate antiviral host-cell responses [4], [5].

Infectious bursal disease virus (IBDV), the best characterized member of the *Birnaviridae* family, is an important avian pathogen with a bipartite dsRNA genome [6]. Available data indicates that IBDV enters susceptible cells by a receptor-mediated endocytosis mechanism [6], and that the replication process is entirely cytoplasmic [7]. Transcription of the largest genome segment (3.2 kb) gives rise to bicistronic mRNAs harboring two partially overlapped open reading frames (ORFs) encoding VP5, a nonstructural polypeptide dispensable for virus replication in tissue culture [8], [9], and a large polyprotein that is self-processed yielding the capsid precursor protein pVP2, the protease VP4, and the VP3 polypeptide [10]. The shorter segment (2.8 kb) contains a single ORF that codes for the VP1 protein acting as RdRp and as a VPg (viral protein genome-linked) primer during RNA synthesis [11].

Birnaviruses represent the only known exception within the realm of dsRNA viruses by encapsidating their dsRNA genomes within a single-layered T = 13 capsid [12], [13]. Instead of the T = 2 transcriptional cores found in the all other icosahedral dsRNA viruses, the inner space of birnavirus T = 13 particles is occupied by filamentous RNPs that are highly reminiscent of replication and transcription complexes found in +ssRNA viruses [14]. RNPs from both IBDV and infectious pancreatic necrosis virus (IPNV), the prototype member of the *Birnaviridae* family, are formed by the dsRNA genome segments covalently linked to the VPg form of VP1, free VP1, and associated to the VP3 polypeptide [15], [16]. VP3 is a multifunctional protein that binds dsRNA [17], [18], acts as a scaffolding protein during capsid assembly, and recruits and activates the RdRp [19]–[21]. VP3 is an anti-apoptotic protein that prevents the activation of the cellular dsRNA-dependent protein kinase (PKR) [22], and it has been recently shown to act as a proficient silencing suppressor in a plant-based recombinant study model [23].

As it is the case for the replication complexes of members of the *Togaviridae* family [24], we have recently shown that IBDV RNPs associate to the cytosolic face of modified endocytic membranes [25]. Demonstration that RNPs isolated from purified IBDV virions are fully competent for RNA synthesis in vitro [16], suggested that IBDV RNP complexes might act as capsid-independent transcriptional engines during virus replication.

In this report, we describe the development of a cell-based IBDV RNP assembly system devoid of the capsid protein that efficiently triggers the expression of IBDV polypeptides and the production of infectious IBDV. These results demonstrate that RNPs assembled in the absence of the capsid polypeptide are fully capable of initiating a productive IBDV replication process. This finding provides new clues for the understanding of the birnavirus replication strategy that differs from that of prototypical dsRNA viruses, and strengthens the hypothesis concerning the existence of an evolutionary link between birnaviruses and +ssRNA viruses.

## Materials and Methods

### Cells and Viruses

The IBDV Soroa strain, a cell-adapted serotype I virus, was propagated in QM7 cells (Quail myoblasts, ATCC number CRL-1962) as previously described [16]. Recombinant vaccinia viruses (VACV) VT7LacOI, VT7-VP1 and VT7-VP3 have been previously described [20], [26]. Cells used in this study were grown in Dulbecco’s modified Eagle’s medium (DMEM) containing 10% fetal calf serum (FCS) at 37°C and 5% CO_2_. VACVs were propagated and titrated in BSC40 cells (African green monkey kidney epithelial cells, ATCC number CRL-2761). QM7 cells were used for the IBDV rescue assays. IBDV growth and titrations were carried out in DF-1 cells (Chicken embryo fibroblasts, ATCC number CRL-12203).

### Purification of Genomic IBDV dsRNA

Genomic dsRNA was extracted from preparations of sucrose gradient-purified IBDV as previously described [16]. Briefly, purified IBDV preparations were incubated with 1% SDS for 3 min at 100°C followed by treatment with proteinase K (2 mg/ml, 1 h, 37°C). The dsRNA was then extracted with TriZol (Invitrogen) and purified using silica-based mini-spin columns (Quiagen). Isolated dsRNA samples were treated with 50 units/µg of dsRNA of RNase T1 (Roche), specifically digesting ssRNA, at 37°C for 30 min to eliminate potentially contaminating ssRNA traces, and then subjected to a second round of purification on mini-spin columns. dsRNA concentrations were determined using a Nanodrop spectrophotometer (Thermo scientific). The effectiveness of the RNase T1 was tested on irrelevant ssRNA samples. For control experiments, RNase T1-treated purified dsRNA was treated with RNase III (Invitrogen), specifically digesting dsRNA, to eliminate long dsRNA molecules. RNA samples were incubated for 1 h at 37°C with 1 U of RNase III, and then purified as described above.

### Transfection of BSC-40 and QM7 Cells Expressing VP1 and VP3 with Purified dsRNA from IBDV Virus

BSC40 monolayers grown in 12-well plates were transfected with 50 ng of IBDV RNase T1-treated purified dsRNA using Lipofectamine 2000 reagent (Invitrogen). RNA and Lipofectamine 2000 were individually diluted in OptiMEM I (Invitrogen) and mixed according to manufacturer’s instructions. Mock-transfections were performed using mixtures of Lipofectamine 2000 reagent and OptiMEM I. Lipofectamin-dsRNA complexes were added to cells that had been infected with VT7LacOI, VT7-VP1, VT7-VP3, or VT7-VP1 and VT7-VP3, and maintained in the presence of 2 mM isopropyl β-D-1-thiogalactopyranoside (IPTG) for 6 h. Before transfection, cells were washed twice with fresh DMEM to eliminate the IPTG.

### Confocal Laser Scanning Microscopy (CLSM) Analysis

Cells seeded onto glass coverslips were subjected to infection with IBDV or with rVACV and transfected with dsRNA. At the indicated times post-infection (p.i.) or p.t. (p.t.), cells were fixed with cold methanol for 5 min at −20°C, and then air dried and blocked in PBS containing 5% FCS for 30 min at room temperature. Thereafter, coverslips were incubated with the indicated combinations of primary antibodies; rat anti-VP1 [20], rabbit anti-VP2 [27], rabbit anti-VP3 [27], rabbit anti-VP4 and mouse anti-dsRNA antisera (K1 monoclonal antibody, English & Scientific Consulting Bt.) diluted in PBS supplemented with 1% FCS for 45 min at 37°C. The anti-VP4 serum, generated in rabbits immunized with the VP4 polypeptide expressed in *E. coli*, was generously provided by JR Castón. Coverslips were repeatedly washed in PBS, and incubated with the appropriate secondary antibody; goat anti-rabbit, -mouse or -rat Ig coupled to Alexa-488 (green), −594 (red) or −647 (far red), respectively, diluted in PBS supplemented with 1% FCS for 45 min at 37°C. Cell nuclei were stained with 2 4′,6-diamidino-2-phenylindole (Dapi; Sigma) diluted in PBS for 30 min at room temperature. Finally, coverslips were dehydrated with ethanol and mounted with ProLong antifade reagent (Invitrogen). Samples were visualized by epifluorescence using a Leica TCS-Sp5 microscope confocal system. Fluorescent signals detected by CLSM were recorded separately by using appropriate filters. Images were captured using the LAS-AF v.2.6.0 software package (Leica Microsystems).

### SDS-PAGE and Western Blotting

Samples used for Western blot analysis were prepared by removing media from cell monolayers and resuspending the cells in ice-chilled disruption buffer (0.5% Triton X-100; 50 mM KCl; 50 mM NaCl; 20 mM Tris-HCl [pH 7.5]; 1 mM EDTA, 10% glycerol; complete protease inhibitor cocktail [Roche]). Cell lysates were mixed (v/v) with 2x Laemmli's sample buffer and heated at 100°C for 5 min. Electrophoreses were performed on 11% polyacrylamide gels. For Western blot analyses, after electrophoresis, proteins were electroblotted onto Hybond-C nitrocellulose membranes. Before incubation with specific antisera, membranes were blocked by incubation with 5% nonfat dry milk in PBS for 1 h at room temperature. Western blots were carried out using rabbit recognizing the IBDV VP1, VP2, VP3, VP4 and the VACV D13 [28] polypeptides, respectively. After incubation with primary antibodies, membranes were thoroughly washed with PBS, incubated with goat anti-rabbit IgG-Peroxidase conjugate (Sigma), and immunoreactive bands detected by chemiluminescence reaction (GE Healthcare).

### IBDV Tritration

Monolayers of QM7 cells grown in 6-well tissue culture plates were infected with serial dilutions of cell supernatants containing IBDV virus. Following virus adsorption for 1 h at 37°C inocula were removed, cells washed twice with DMEM, and then incubated with fresh DMEM containing 2% of FCS at 37°C in a 5% CO_2_ atmosphere. At 48 h p.i., cells were fixed in acetone-methanol (1∶1), washed with PBS, and incubated at room temperature for 90 min with PBS supplemented with 5% nonfat dry milk in PBS. Thereafter, cells were incubated with a rabbit anti-VP2 antibody diluted in PBS supplemented with 5% nonfat dry milk for 90 min at room temperature. After extensive washing with PBS, monolayers were incubated with horseradish-peroxidase-conjugated polyclonal goat anti-rabbit antibody (GE Healthcare) for 90 min at room temperature. After washing with PBS, antibody-labeled cells were developed using 3, 3′-diaminobenzidine (Sigma) substrate solution, foci of stained cells were counted, and virus titers calculated.

## Results

### The VP2 Capsid Protein does not Co-localize with VP1, VP3 or dsRNA (RNPs) at Early Times Post-infection

In order to determine whether the assembly of IBDV RNPs might take place independently from the virus capsid, we analyzed the subcellular distribution of viral proteins and dsRNA at different times p.i. Preliminar experiments showed that under the conditions used for our analyses, accumulation of virus-encoded proteins begins to be clearly detectable in a significant number of cells at 8 h p.i. (supporting Fig. S1). Accordingly, IBDV-infected DF-1 cells were fixed at 8, 15 or 22 h p.i., respectively. Samples were used for CLSM analysis after incubation with different combinations of antibodies specifically recognizing the VP1, VP2 and VP3 polypeptides and dsRNA, respectively.

**Figure 1:**
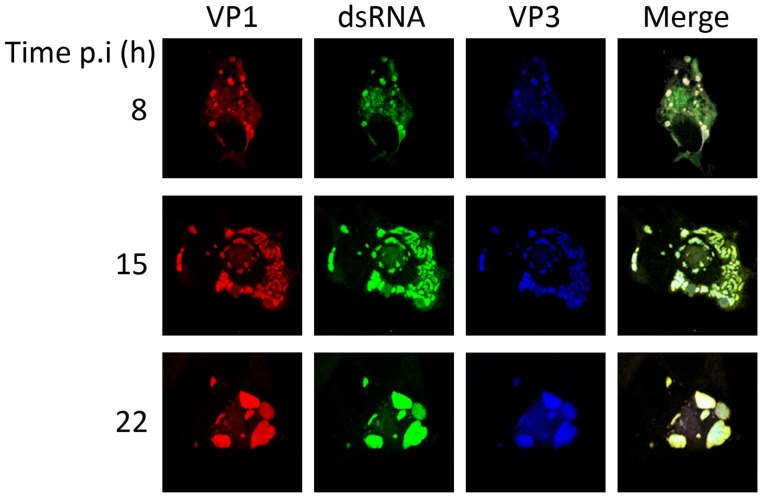
Subcellular localization of IBDV ribonucleoprotein complex components. DF-1 cells were infected with 5 pfu/cell of IBDV. Cells were fixed at 8, 15 or 22 h p.i., respectively, and processed for CLSM using specific antibodies against VP1 (red), dsRNA (green), and VP3 (blue). Fluorescence signals were recorded separately by using appropriate filters. Rightmost panels (Merge) show the overlay of the three fluorescence signals.

Fig. 1 shows representative images of single cells fixed at the selected times p.i. and stained with antibodies against the three RNP components, namely VP1, VP3 and dsRNA. Lower magnification images corresponding to fields of infected cells stained with anti-VP1 and -VP3 antibodies are shown in supporting Fig. S2. The results of this analysis showed that, in the large majority (>90%) of the analyzed cells, the RNP components exhibit a high degree of colocalization throughout the infection process. This analysis also revealed that the RNP immunofluorescence (IF) signal is rearranged during the infection. At early times p.i. (8 and 15 h) RNP complexes appear as numerous granule-like accretions surrounded by a fainter signal of nebulous aspect whilst at the late phase of the infection (22 h p.i.) the RNP components are found in large aggregates (Fig. 1B).

**Figure 2:**
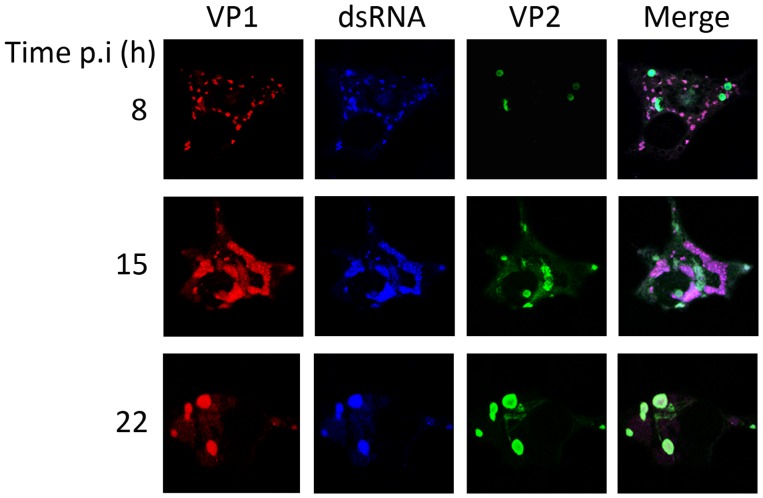
Subcellular localization of IBDV ribonucleoprotein complexes and the VP2 capsid polypeptide. DF-1 cells were infected with 5 pfu/cell of IBDV. Cells were fixed at 8, 15 or 22 h p.i., respectively, and processed for CLSM using specific antibodies against the RNP VP1 (red) and dsRNA (blue) components, and the VP2 capsid polypeptide (green). Fluorescence signals were recorded separately by using appropriate filters. Rightmost panels (Merge) show the overlay of the three signals.

Fig. 2 shows representative images of single cells stained with antibodies against the VP2 capsid polypeptide and two RNP components, namely VP1 and dsRNA. Lower magnification images showing fields of infected cells stained with anti-VP2 and -VP1 antibodies are shown in supporting Fig. S3. At early times p.i. (8 and 15 h), over 80% of the cells showed a VP2 IF signal corresponding to aggregates not overlapping the granular VP1 and dsRNA signals. In addition to VP2 aggregates, a tenuous VP2 signal surrounding and/or overlapping that corresponding to RNP granules was also found at early times p.i. (Fig. 2 and S2). Similarly to what had been observed with RNP components, the VP2 subcellular distribution also undergoes reorganization during the course of infection, and at 22 h p.i. most cells (>80%) showed the presence of VP2 in large aggregates overlapping the RNP-specific signal (Fig. 2 and S3).

Taken together, the described CLSM data suggest that RNPs are assembled early after infection in a process that appears to be largely independent from the capsid polypeptide. Interestingly, at late times p.i. (22 h) the RNP- and VP2-specific signals merge into large aggregates (Figs. 1, 2, S2 and S3) resembling previously described IBDV viroplasms thought to represent virus assembly sites [20].

### Intracellular Assembly of Recombinant IBDV RNPs

Results described above evoked the possibility that RNP complexes detected at early times p.i. might be responsible for genome transcription. However, direct experimental evidence supporting this assumption was lacking. This prompted us to design an experimental approach to test this hypothesis.

We had previously shown that, in the absence of other IBDV protein and genome elements, coexpression of VP1 and VP3 using VACV recombinants leads to the assembly of VP1/VP3 complexes that accumulate within the cell cytoplasm [20]. Based on this observation, we sought to develop an experimental strategy to try and establish a cell-based system to assemble IBDV RNPs devoid of the VP2 capsid polypeptide. For this, cells would be coinfected with two previously described rVACVs, namely VT7-VP1 and VT7-VP3, inducibly expressing the IBDV VP1 and VP3 polypeptides, respectively [20], [26]. Addition of IPTG to the medium would lead to the simultaneous expression of VP1 and VP3. The third RNP component would be supplied “in trans” by transfecting cells with genomic dsRNA isolated from purified IBDV. The effectiveness of this experimental approach could be readily tested by assessing the presence of IBDV proteins other than those expressed by VT7-VP1 and VT7-VP3 recombinant viruses, i.e. the VP4 and VP2 polypeptides, in cell cultures.

**Figure 3:**
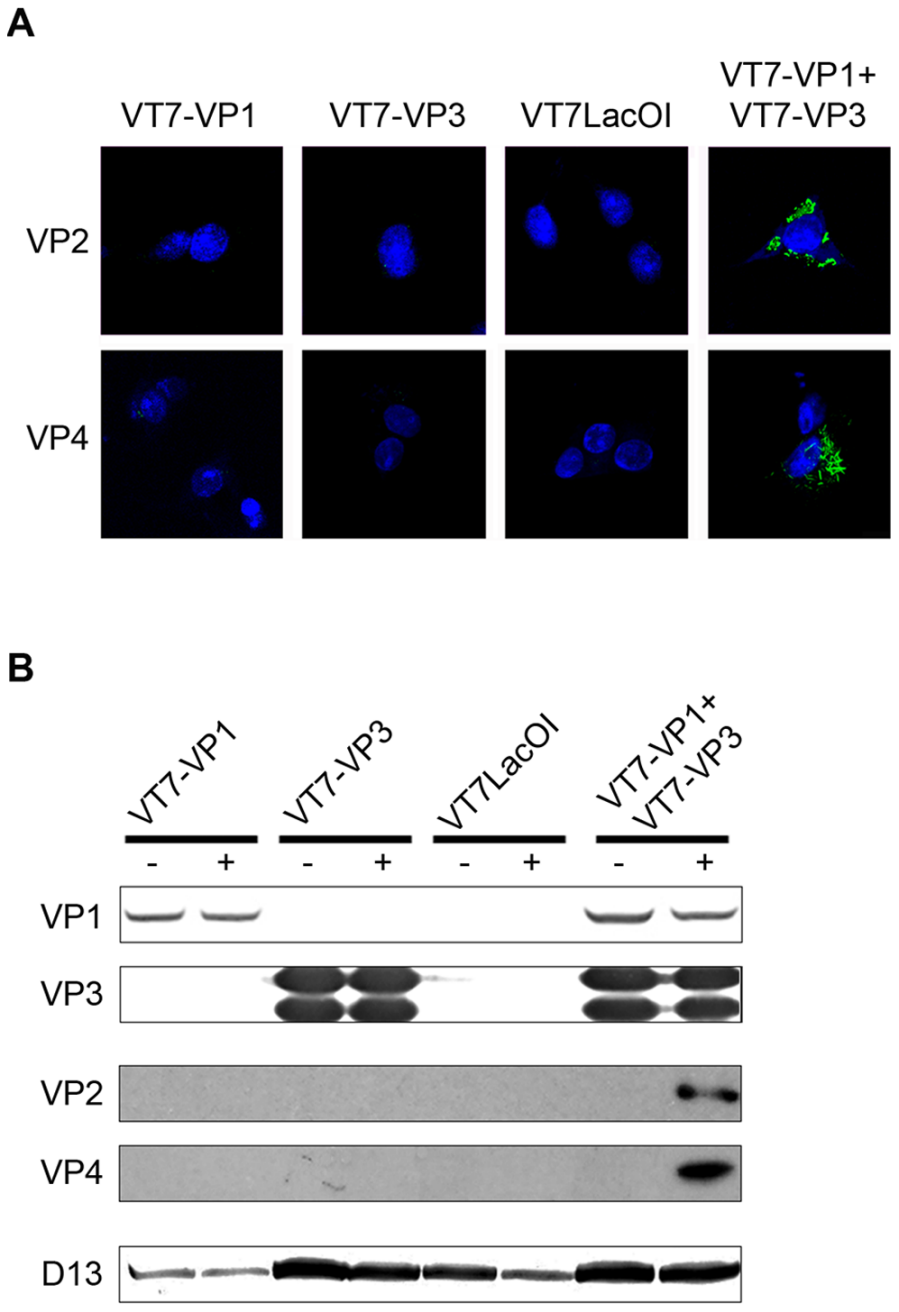
Detection of IBDV-specific protein expressed from synthetically assembled IBDV RNPs. (A) Detection of VP2 and VP4 polypeptides by indirect IF. BSC40 cells were infected with VT7-VP1, VT7-VP3 or VT7LacOI, or coinfected with VT7-VP1 and VT7-VP3, and then incubated with 2% FCS-DMEM containing 2 mM IPTG to induce recombinant protein expression. 6 h after induction, cells were transfected with 50 ng of RNase T1-treated IBDV dsRNA. At 24 h p.t. cells were fixed and processed for CLSM using specific antibodies against VP2 (VP2) or VP4 (VP4), respectively. Cell nuclei were stained with Dapi (blue). (B) Detection of IBDV encoded polypeptides by Western blot. Cell cultures infected as described above were either mock-transfected (−) or transfected (+) with IBDV dsRNA. At 24 h p.t. cells were harvested and the corresponding extracts used for Western blot analysis using antibodies against IBDV VP1, VP2, VP3 and VP4 polypeptides. A blot using serum against the VACV D13 protein was also carried out as a control to assess both VACV infection and protein loading.

The described strategy was tested by infecting BSC40 monolayers with VT7LacOI (the parental VACV used for the generation of the VT7/VP1 and VT7/VP3 recombinant viruses), VT7-VP1 or VT7-VP3 alone or coinfected with VT7-VP1 and VT7-VP3. Infections were performed with 2 PFU/cell of each virus. After infection, cultures were maintained in medium supplemented with IPTG for 6 h. After this period, cultures were washed, either mock-transfected or transfected with RNase T1-treated purified IBDV dsRNA, and then maintained in the absence of IPTG. At 24 h p.t., cultures were either fixed for IF studies or harvested to produce extracts for Western blot analysis. As shown in Fig. 3A, expression of VP2 and VP4 IBDV polypeptides was exclusively detected in cells coinfected with VT7-VP1 and VT7-VP3 and transfected with IBDV dsRNA. The presence of IBDV-encoded polypeptides in extracts from the different cultures was also analyzed by Western blot analysis. Membranes were incubated with anti-VP1, -VP2, -VP3 and -VP4 antibodies, respectively. Additionally, incubation with a serum recognizing the VACV-encoded D13 polypeptide was also included in order to assess both VACV replication and protein loading. In agreement with IF results, expression of the VP2 and VP4 polypeptides was only detected in extracts from cultures coinfected with VT7-VP1 and VT7-VP3 and transfected with IBDV dsRNA (Fig. 3B). Again, these two polypeptides were not detected in samples of cells infected with VT7-VP1, VT7-VP3 or VT7LacOI alone or in mock-transfected cells. As expected, VP1 and VP3 expression was detected in samples from cells infected with their corresponding recombinant VACV. As previously reported, VP3 expression led to accumulation of two immunoreactive bands of slightly different electrophoretic mobility [26].

To further confirm the results described above, three experiments were carried out by transfecting VP1/VP3-expressing cells with purified RNase T1-treated IBDV dsRNA following treatment with RNase III. These experiments rendered negative results, thus ruling out the possibility that the expression of the VP2 and VP4 polypeptides detected in cells transfected with RNase T1-treated IBDV dsRNA might be due to traces of IBDV-derived mRNAs remaining in dsRNA preparations. Similarly, experiments performed in cells coinfected with VT7-VP1 and VT7-VP3 and maintained in the absence of IPTG did not result in VP2 or VP4 expression (data not shown).

Results presented here unambiguously show that complexes formed upon coexpression of the VP1 and VP3 polypeptides provide a functional platform for the transcription of exogenously provided IBDV dsRNA genome segments in the complete absence of the IBDV capsid protein.

### Rescue of Infectious IBDV from Recombinant Transcriptional Complexes

In view of results described above, it was important to assess whether the described IBDV transcriptional platform might also result in the assembly of infectious IBDV. For this, we followed the strategy described above replacing the BSC40 cells for an avian cell line, QM7, highly susceptible to IBDV replication. QM7 cells were either coinfected with VT7-VP1 and VT7-VP3 or mock-infected. After infection, cultures were incubated in the presence of 2% FCS and 2 mM IPTG for 6 h, and then either mock-transfected or transfected with 50 ng of RNase T1-treated IBDV genomic dsRNA. At 48 h p.t., culture media were harvested and cell debris eliminated by low speed centrifugation. Clarified supernatants were passed through 0.1 µm filters to eliminate VACV particles. The resulting samples were treated with RNAse III and RNAse T1 to eliminate potential dsRNA and ssRNA contaminants, and then used to determine the presence of infectious IBDV by incubating fresh QM7 cell monolayers with neat or 10^−1^ dilutions of the filtrates for 96 h. Thereafter, cells were harvested and used to search for the presence of IBDV polypeptides by Western blot analysis. Additionally, the corresponding cell media were collected and used to perform IBDV titrations.

To detect IBDV-encoded polypeptides, Western blot analyses were carried out with anti-VP1, -VP2, -VP3 and -VP4 sera. Additionally, the possible presence of infectious VACV potentially contaminating the analyzed samples was assessed by Western blot using a serum specific for the VACV D13 protein.

**Figure 4:**
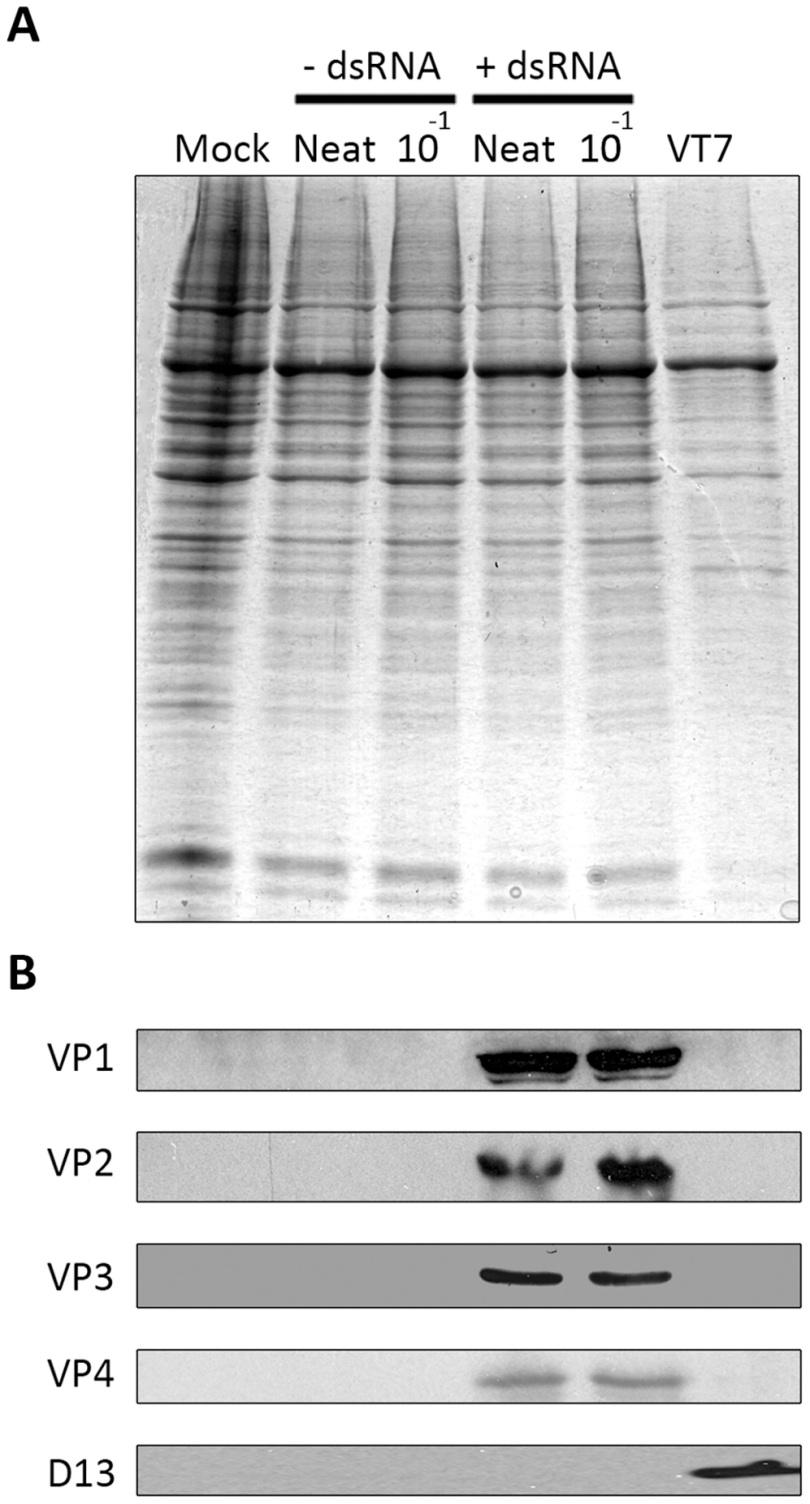
Rescue of infectious IBDV from synthetically assembled RNPs. Cell media collected at 48-VP1 and VT7-VP3, either mock-transfected (−dsRNA) or transfected (+dsRNA) with purified genomic IBDV dsRNA, were passed through 0.1 µm filters to eliminate VACV particles and then treated with RNase T1 and RNase III to digest potential ss and dsRNA contaminants. Fresh DF-1 monolayers were incubated with either neat or a 10^−1^ dilution of the filtrates for 1 h. Thereafter, monolayers were washed twice with fresh medium and maintained in DMEM with 2% FCS. At 96 h p.i. cells were harvested and the corresponding extracts used for **(A)** SDS-PAGE analysis followed by Coomassie blue staining, and **(B)** Western blotting using sera against the IBDV VP1, VP2, VP3 and VP4 polypeptides, and the VACV D13 protein. An extract from VT7-infected (VT7) cells was included as a positive control to assess the reactivity of the anti-D13 serum.

As shown in Fig. 4, IBDV-derived immnuoreactive proteins were not found in cells incubated with samples from cells coinfected with VT7-VP1 and VT7-VP3 and mock-transfected with dsRNA. In contrast, all four tested IBDV proteins were detected in extracts from cells incubated with samples from cultures coinfected with VT7-VP1 and VT7-VP3 and transfected with IBDV dsRNA. The D13 polypeptide was only detected in samples from VT7-infected cells, thus ruling out the presence of contaminating VACV in the analyzed samples.

In agreement with Western blot data, supernatants collected from cells incubated with filtrates from cultures coinfected with VT7-VP1 and VT7-VP3 and transfected with IBDV dsRNA exhibited IBDV titers nearing 10^8^ PFU/ml, akin to those obtained when the Soroa strain is propagated in QM7 cells at low MOI. Infectious IBDV was not detected in samples from mock-transfected cells. The experiment was independently repeated three times with similar results.

Data described here unambiguously demonstrate that the activity of the developed recombinant transcriptional complexes efficiently triggers the assembly of infectious IBDV.

## Discussion

The characterization of IBDV has resulted in a number of unexpected structural and functional findings. The demonstration that IBDV particles lack the internal T = 2 transcriptional core ubiquitously found in icosahedral dsRNA viruses provided a clear structural signature differentiating birnaviruses from the rest of dsRNA viruses [12]. Subsequent studies showed that birnavirus T = 13 capsids enclose filamentous RNP complexes formed by the genomic dsRNA segments, the RdRp in both its VPg and “free” form, and the RNA-binding VP3 protein [15], [16]. This finding further strengthened the notion that the birnaviral replication cycle might exhibit profound differences with respect to that of prototypical dsRNA viruses. However, experimental evidences outlining these expected differences were as yet lacking. Results described in this report address one of the major pending questions: whether birnaviral RNP complexes act as independent transcriptional entities during virus replication.

Although CLSM data gathered during IBDV infection indicated that RNPs might act as independent transcriptional complexes, a definitive answer for this question was pending. To tackle this experimental issue we decided to use a direct approach based on the use of VP1/VP3 complexes preformed in the cytoplasm of cells. As described above, we had previously shown that VP1 and VP3 form intracellular complexes following coexpression of both polypeptides from inducible recombinant VACVs [20]. This expression system allows the production of high levels of VP1/VP3 complexes in the absence of the capsid polypeptide, thus providing an excellent starting point to generate IBDV RNPs completely devoid of the virus capsid. The third RNP component, the dsRNA genome, was provided *in trans* by transfecting VP1/VP3-expressing cells with highly purified IBDV genomic dsRNA. Indeed, to avoid misleading results, utmost care was taken to prevent the presence of IBDV-derived ssRNA potentially contaminating the dsRNA preparations. Hence, the preparation of the IBDV dsRNA samples used in this report included an exhaustive treatment with RNase T1, an endoribonuclease that specifically degrades ssRNA [29], followed by a second round of RNA purification.

CLSM and Western blot data conclusively show that the presence of the three RNP elements is necessary and sufficient to trigger the expression of proteins encoded within the exogenously provided IBDV genome segments. As predicted, the absence of any of these elements rendered negative results. The specificity of the described IBDV-rescue system was further confirmed by the negative results obtained in control experiments performed with dsRNA samples treated with RNase III that specifically degrades long dsRNA molecules [30]. The capability of the recombinant RNPs to rescue infectious IBDV evidences that, in the absence of the virus capsid, RNP complexes are competent for initiating of a productive IBDV infection.

Indeed, the RNP assembly system described here might be also useful to analyze the specific role of VP1 and VP3 domains for RNA transcription thus providing a useful alternative to overcome certain limitations of currently used IBDV reverse genetics approaches [31].

Attempts to reproduce the described results using conventional virus-free eukaryotic expression vectors have been, as yet, unsuccessful. A possible explanation for this might lay in the fact that the expression levels of the VP1 and VP3 achieved with such vectors are much lower than those obtained using VACV recombinants, thus strongly reducing the number of available VP1/VP3 complexes and the chances of assembling active RNPs. Additionally, we have observed that transfection of IBDV dsRNA to cultures previously transfected with eukaryotic vectors expressing the VP1 and VP3 polypeptides results in an extensive apoptotic response and the subsequent destruction of cell monolayers, hence further reducing the likelihood of generating active RNP complexes under such experimental conditions.

Evidence that IBDV artificially assembled RNPs are capable of transcribing the dsRNA genome in the absence of the T = 13 virus capsid provides additional support for the recently proposed birnavirus replication model that entails the disassembly of the incoming virus particles followed by the release of the virion-enclosed RNPs from endosomes used during the virus entry process [25].

The fact that the pVP2 and VP3 polypeptides are released from the same polyprotein precursor intuitively suggests the possibility that the assembly of pVP2/VP3 complexes might be produced immediately after polyprotein processing. However, in addition to interacting with the pVP2, VP3 must also interact with both the dsRNA genome and the RdRp [17], [20], [21] to assemble RNP complexes. The success of the replication process likely involves a complex set of timely-regulated interactions concerning all the structural IBDV polypeptides. In this context, it seems likely that RNPs either released from infecting virions or assembled at the beginning of the infection process might be preferentially devoted to mRNA synthesis rather than to virus assembly. This hypothesis is consistent with CLSM data presented here showing that early after infection the three RNP components (VP1, VP3 and dsRNA) merge within discrete granules largely devoid of the VP2 polypeptide.

### Evolutionary Insigths

The presence of IBDV structural elements reminiscent of +ssRNA was first recognized by Caulibaly et al. [32]. In their report describing the crystal structure of the VP2 polypeptide, these authors revealed that whilst the VP2 protruding domain exhibits a high degree of structural homology to its counterparts from the reovirus T = 13 capsid protein, the VP2 shell domain is akin to those building the T = 3 capsid of the +ssRNA nodaviruses. Interestingly, the parallelism between +ssRNA nodaviruses and birnaviruses extend beyond this structural aspect, i.e. the maturation of their capsid polypeptides involves the self-processing of the precursor capsid protein [33], and their entry mechanisms rely upon capsid-associated amphipathic peptides [34]. Birnaviruses share other important biological features with +ssRNA viruses, i.e. the non-canonical organization of the RdRp palm domains (exclusively found in birnaviruses, members of the *Permutotetraviridae,* and the alpha-like dubbed Grapevine virus Q (GVQ) [35]), and the protein-primed mechanism of RNA transcription, akin to that used by picornaviruses [36]. All these similarities provided the basis to propose that birnaviruses might represent an evolutionary cross-road between dsRNA and +ssRNA viruses [14], [32], [37]. Results described here provide a functional support for this hypothesis.

## Supporting Information

Figure S1 **Detection of the VP3 polypeptide in IBDV-infected cells.** DF-1 cells were infected with 5 pfu/cell of IBDV. Cells were fixed at 4, 8, 15 or 22 h p.i., respectively, and processed for CLSM using specific antibodies against VP3 (green). Cell nuclei were stained with Dapi (blue). Fluorescence signals were recorded separately by using appropriate filters. Images show the overlay of both fluorescence signals.
(TIF)

Figure S2 **Subcellular localization of IBDV ribonucleoprotein complex components.** DF-1 cells were infected with 5 pfu/cell of IBDV. Cells were fixed at 8, 15 or 22 h p.i., respectively, and processed for CLSM using specific antibodies against VP1 (red), and VP3 (green). Cell nuclei were stained with Dapi (blue). Fluorescence signals were recorded separately by using appropriate filters. Rightmost panels (Merge) show the overlay of the three fluorescence signals.
(TIF)

Figure S3 **Subcellular localization of IBDV ribonucleoprotein complex components.** DF-1 cells were infected with 5 pfu/cell of IBDV. Cells were fixed at 8, 15 or 22 h p.i., respectively, and processed for CLSM using specific antibodies against VP1 (red), and VP2 (green). Cell nuclei were stained with Dapi (blue). Fluorescence signals were recorded separately by using appropriate filters. Rightmost panels (Merge) show the overlay of the three fluorescence signals.
(TIF)
