# Supplementary figures and images for: Rescue of Infectious Birnavirus from Recombinant Ribonucleoprotein Complexes
Source: PLoS One. 2014 Jan 30;9(1):e87790. doi: 10.1371/journal.pone.0087790 (PMC3907549; doi:10.1371/journal.pone.0087790)

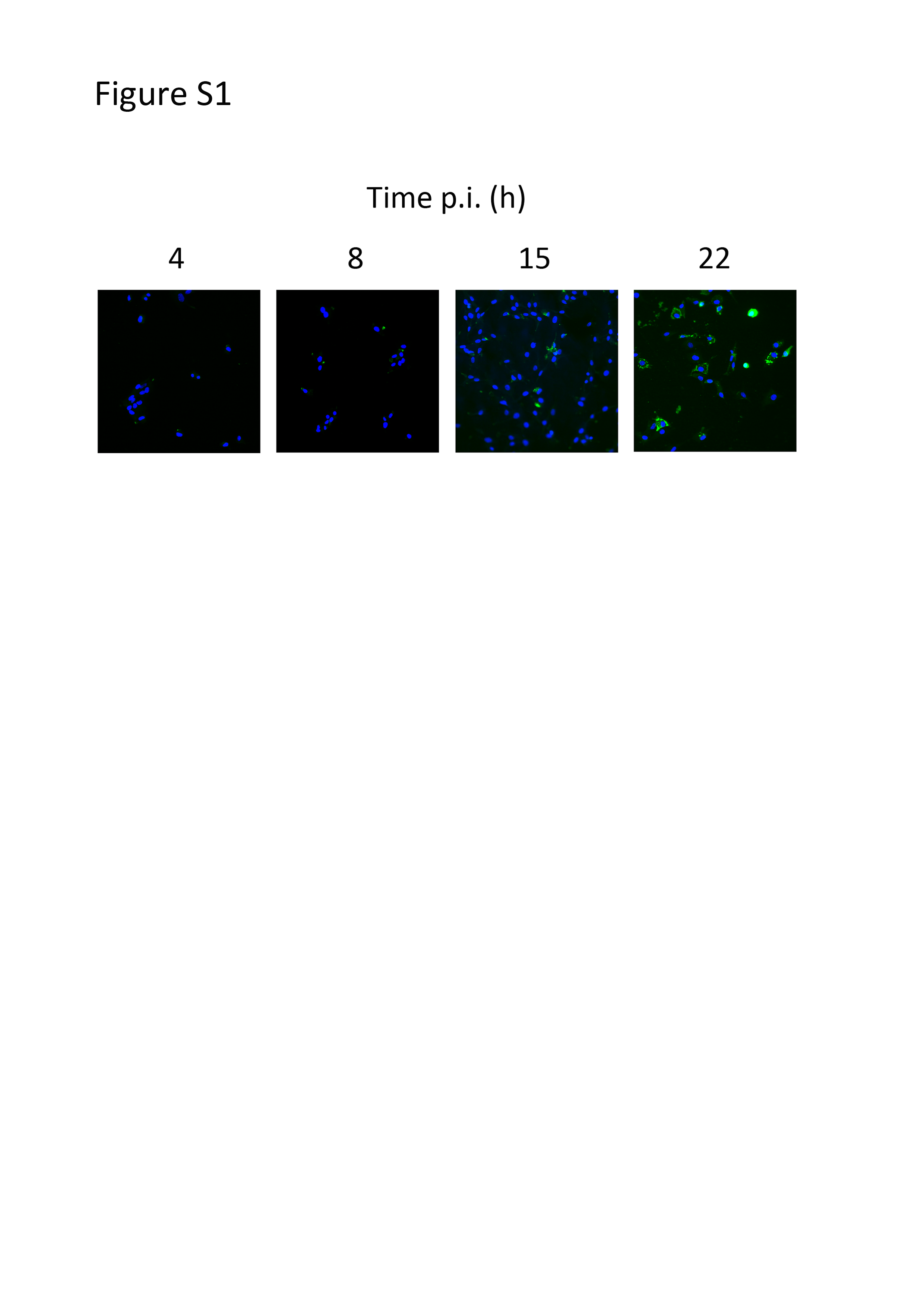

Supplement: Figure S1 — Detection of the VP3 polypeptide in IBDV-infected cells. DF-1 cells were infected with 5 pfu/cell of IBDV. Cells were fixed at 4, 8, 15 or 22 h p.i., respectively, and processed for CLSM using specific antibodies against VP3 (green). Cell nuclei were stained with Dapi (blue). Fluorescence signals were recorded separately by using appropriate filters. Images show the overlay of both fluorescence signals. (TIF) [file pone.0087790.s001.tif]

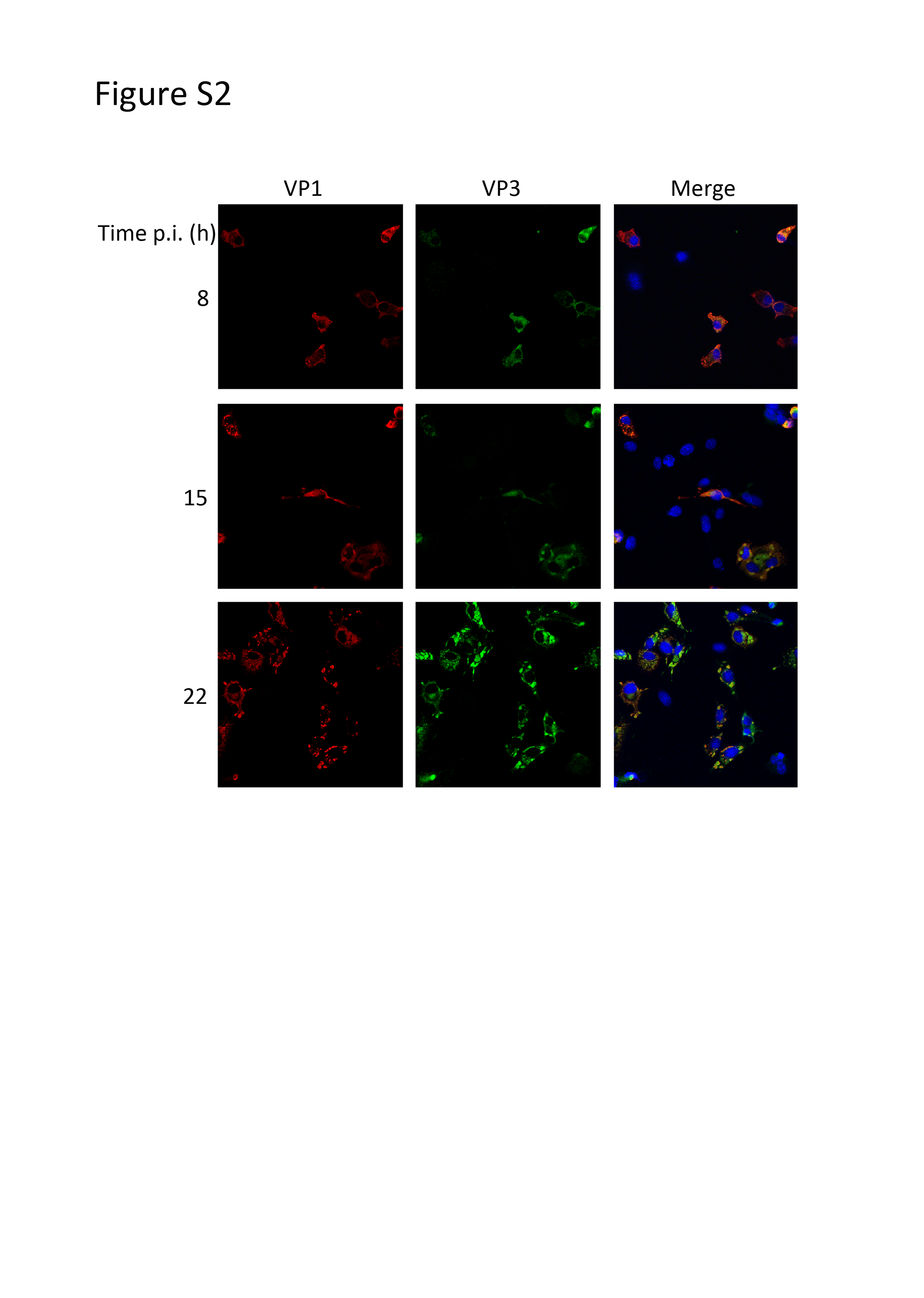

Supplement: Figure S2 — Subcellular localization of IBDV ribonucleoprotein complex components. DF-1 cells were infected with 5 pfu/cell of IBDV. Cells were fixed at 8, 15 or 22 h p.i., respectively, and processed for CLSM using specific antibodies against VP1 (red), and VP3 (green). Cell nuclei were stained with Dapi (blue). Fluorescence signals were recorded separately by using appropriate filters. Rightmost panels (Merge) show the overlay of the three fluorescence signals. (TIF) [file pone.0087790.s002.tif]

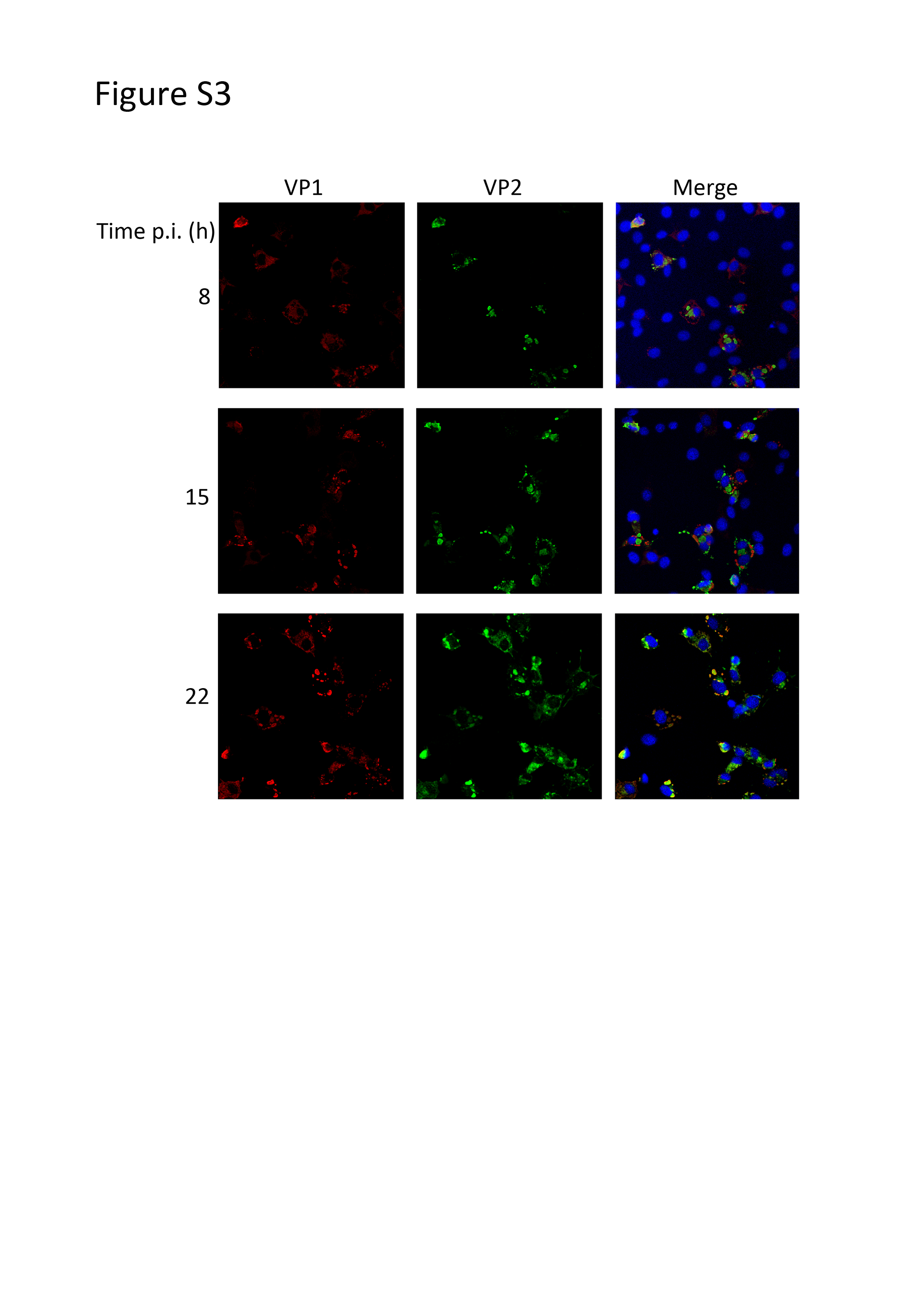

Supplement: Figure S3 — Subcellular localization of IBDV ribonucleoprotein complex components. DF-1 cells were infected with 5 pfu/cell of IBDV. Cells were fixed at 8, 15 or 22 h p.i., respectively, and processed for CLSM using specific antibodies against VP1 (red), and VP2 (green). Cell nuclei were stained with Dapi (blue). Fluorescence signals were recorded separately by using appropriate filters. Rightmost panels (Merge) show the overlay of the three fluorescence signals. (TIF) [file pone.0087790.s003.tif]
